# Mental Health and Drug Use Patterns Among Men Who Have Sex with Men (MSM) Engaging in Chemsex in the UK

**DOI:** 10.3390/healthcare13070719

**Published:** 2025-03-24

**Authors:** Lukasz Lagojda, Deberson Ferreira de Jesus, Daniel Kinnair, Marc Chrysanthou

**Affiliations:** 1 Sheffield Centre for Health and Related Research (SCHARR), School of Medicine and Population Health, University of Sheffield, Sheffield S1 4DA, UK; 2 Warwickshire Institute for the Study of Diabetes, Endocrinology and Metabolism (WISDEM), University Hospitals Coventry & Warwickshire NHS Trust, Coventry CV2 2DX, UK; 3 School of Health and Society, Faculty of Education, Health and Wellbeing, University of Wolverhampton, Wolverhampton WV1 1LY, UK; m.chrysanthou@wlv.ac.uk; 4 Núcleo de Estudos e Pesquisas Sobre as Relações de Gênero, Federal University of Mato Grosso, Cuiaba 78060-900, Brazil; deberson.jesus@ufmt.br; 5 George Davies Centre, Leicester Medical School, University of Leicester, Lancaster Road, Leicester LE1 7HA, UK; daniel.kinnair@nhs.net

**Keywords:** mental health, Chemsex, substance use, addiction

## Abstract

**Background:** Chemsex is a subtype of sexualized drug use which is increasingly more prevalent amongst gay, bisexual, and other men who have sex with men (MSM). This use of psychoactive substances in sexual contexts poses an emerging public health concern, since it has been associated with an array of health risks, including mental health risks. **Objective**: As Chemsex appears to be increasing in the UK, this study aimed to explore Chemsex patterns and mental health amongst Chemsex users in the UK. **Methods:** Chemsex/drug use patterns and the prevalence of coexisting depression and anxiety, based on the Hospital Anxiety and Depression Scale (HADS), were assessed in an MSM sample via an anonymous online survey between December 2023 and February 2024. **Results:** A total of 141 UK adults participated in the survey (age range: 26–41 years). High overall use of the three common Chemsex-related substances was reported, including γ-hydroxybutyrate/γ-butyrolactone (GHB/GBL) (77.3%), synthetic cathinones (64.5%), and methamphetamine (47.5%). Injecting (“slamming”) was less prevalent (17.0%), although this practice was the most common amongst weekly Chemsex users (54.2%). Based on provided HADS responses, a high incidence of anxiety (49.9%) was reported among the study population, with the highest (abnormal) anxiety scores observed among weekly Chemsex users (44.4%). The incidence of depression was lower overall (27.0%), whilst the highest (abnormal) scores were also observed in weekly Chemsex users (61.5%). **Conclusions:** As relevant UK data remain limited, this study offers novel insight into Chemsex patterns and mental health challenges among MSM in the UK, suggesting unmet needs for targeted interventions on mental health issues among this population. Further large-scale and prospective studies are necessary to explore the nuanced interplay between the frequency of Chemsex use and its impact on mental health.

## 1. Introduction

The term “Chemsex” is an amalgamation of “chemical” and “sex”, which is used to describe the intentional use of psychoactive substances before or during planned sexual activity [1]. The main substances used during Chemsex include γ-hydroxybutyrate (GHB) and/or its precursor γ-butyrolactone (GBL), crystal methamphetamine, and synthetic cathinones [2]. While most authors limit Chemsex definition to instances where the three substances are consumed, a broader spectrum of recreational drugs has been reported, including ketamine, cocaine, and infrequently, amphetamines, ecstasy/methylenedioxymethamphetamine (MDMA), or cannabis [3]. Additionally, the use of erectile dysfunction medications and alcohol alongside alkyl nitrates (i.e., “poppers”) has also been documented in the context of Chemsex, emphasizing the polydrug nature of this practice [2,4,5,6].

Despite the absence of a universally accepted definition, Chemsex is prominent among gay, bisexual, and other MSM [7,8]. In a comprehensive systematic review synthesizing data from 38 studies published in the period between 2010 and 2017, the estimated prevalence ranged between 3.6% and 93.7% depending on the Chemsex setting and its definition [9]. The prevalence of Chemsex also exhibits a marked variability in the UK, where, during the same temporal period, engagement in Chemsex ranged from 17% among MSM attending sexual health centres to 31% among HIV-seropositive MSM attending a single clinic in London [10], whereas a recent cross-sectional study conducted in London during a seven-day period of the COVID-19 pandemic in August 2020 reported a Chemsex prevalence of 14.1% [11,12].

Chemsex has increased in popularity over the past few decades, becoming a significant public health concern [13]. The harms associated with Chemsex are multifaceted and pose significant negative impacts on physical health [14], social functioning [15], and mental wellbeing [16,17]. GHB/GBL is a potent sedative at higher doses, although it can produce a “mild high” and a stimulating effect at lower doses [18], whereas both methamphetamine and synthetic cathinones are potent stimulants [19,20]. In combination, these substances increase disinhibition, facilitating sexual compulsions that may lead to an intense fixation on sexual desires and an inability to restrain them, resulting in hypersexuality [21,22,23]. Indeed, Chemsex users agree that this practice often serves to initiate, extend, sustain, and intensify sexual encounters [24]. This carries a risk of increased engagement in risky sexual behaviours, including condomless anal intercourse with multiple sexual partners, thereby increasing the risk of contracting sexually transmitted infections [25]. Additionally, Chemsex substances can lead to severe physical health problems, including cardiovascular complications [26,27], psychiatric manifestations [28,29], and death [30,31]. Socially, Chemsex can strain relationships with partners, family, and friends, and it can also lead to financial difficulties, legal problems, and disruptions to employment or education [32].

Amongst the most critical concerns associated with Chemsex is its profound negative impact on mental wellbeing [16,33]. Growing research suggests high rates of psychological distress among Chemsex users, with data from systematic reviews and meta-analyses indicating that those engaging in Chemsex are significantly more likely to experience symptoms of anxiety and depression compared to those who do not engage in Chemsex [16,17]. For example, an early study conducted amongst MSM in London and Brighton showed that Chemsex was significantly associated with clinically significant symptoms of anxiety and depression (prevalence ratio, PR = 1.49, 95% confidence interval, 95% CI = 1.10 to 2.01 and PR = 1.47, 95% CI = 1.08 to 2.00 for anxiety and depression, respectively) [16,34]. Additionally, a Norwegian study conducted amongst MSM attending sexual health clinics reported that men who engaged in Chemsex had two times greater odds of reduced mental health compared to those who did not report using drugs during sex (adjusted odd ratio, OR =  2.18, 95% confidence interval, 95% CI =  1.25 to 3.78) [16,35]. Furthermore, a 2020 study among 1050 MSM conducted in Germany showed that 11.9% and 8.3% of those who reported engaging in Chemsex had clinically significant depressive and anxiety symptoms, respectively [16,36]. Interestingly, a longitudinal study involving non-intravenous Chemsex users in Belgium reported that 12% of the cohort exhibited moderate-to-severe depression levels at baseline [16,37]. This proportion increased to 15% and 18% at 9 and 18 months, respectively, supporting the notion of a dose-response relationship between the frequency of substance use and the deterioration of mental health [37]. More recently, in a sample of 78 MSM who self-referred to a substance use service in Paris, France, 19% and 42% presented with a depressive or anxiety disorder, respectively [38]. Additionally, in a cross-sectional study amongst 359 MSM living with HIV in the Netherlands, 47.8% and 27.1% of those engaging in Chemsex presented with potential anxiety or clinical depression, respectively [39].

In addition to non-intravenous drug use in Chemsex contexts, combining Chemsex with injecting, or Slamsex (‘slamming’ colloquially describes the practice of intravenously injecting drugs, such as crystal methamphetamine, in a sexual setting), has been associated with even greater mental health risks, including severe anxiety, depression, and suicidal behaviours [40,41,42]. For example, in a 2023 cross-sectional study involving 230 Chemsex users in Spain, Slamsex was associated with an increased risk of anxiety or depression [40]. Additionally, those injecting drugs were more likely to experience suicidal ideation, psychosis, and exhibit suicidal behaviours compared to those engaging in non-intravenous use [40]. Additionally, an investigation conducted across five French cities showed that Slamsex participants exhibited notably diminished mental health, as evidenced by a mean score 15 points lower on a 100-point scale when compared to those who abstained [41]. Furthermore, a study conducted in the Netherlands reported that over three-quarters (76%) of the 175 study participants experienced life-long psychological problems because of Slamsex, including insomnia, depressive feelings, and anxiety or panic attacks [42].

While the existing literature highlights the harmful impact of Chemsex on mental health, much of this evidence is derived from secondary data collected primarily in sexual health clinics or from broader recruitment settings [43]. Therefore, there is a paucity of dedicated investigations focusing on the mental health outcomes of Chemsex users, which limits our understanding of the mental health status of MSM engaging in Chemsex. Accordingly, the aim of the present study is to explore Chemsex patterns and mental health amongst a sample of Chemsex users residing in the UK.

## 2. Materials and Methods

This study was conducted as part of a master’s in public health degree undertaken by the lead author (LL) at the University of Wolverhampton. All necessary ethical approvals were granted by the host institution before data collection commenced. Participation in the study was incentivized by the possibility of voluntarily entering a prize drawing that consisted of a single GBP 50 Amazon voucher.

### 2.1. Study Design

The reporting of this study was carried out in adherence with the Strengthening the Reporting of Observational Studies in Epidemiology (STROBE) recommendations for reporting observational studies [44]. This study used a cross-sectional online survey to investigate anxiety and depression in a sample of MSM who actively participated in Chemsex. The survey instrument consisted of an informed consent page followed by a 24-item questionnaire that was structured into three sections relating to mental health, Chemsex behaviours/drug use patterns, and demographic characteristics. After completing the survey, participants were offered voluntary entry into a prize drawing. For this purpose, a separate form was created to collect contact information, which was made available via a link upon completion of the main survey.

### 2.2. Inclusion Criteria and Sampling Procedure

The specific inclusion criteria were the following: UK adults (age: ≥18 years); gay, bisexual, or other MSM; and must practice Chemsex. The main exclusion criteria included individuals who did not consent to participate in the survey; non-UK residents; and those whose English language skills were not sufficient to understand the study scope and the survey questionnaire. Due to the sensitive nature and specificity of the target population, non-probability sampling was selected. As opposed to probability-based approaches, which employ random, systematic, stratified, or cluster techniques to sample across the general population, non-probability techniques seek samples that are immediately and easily available to the researcher [45,46]. For this purpose, a “snowball” sampling method was used, as this approach is effective at assessing individuals that are otherwise difficult to reach [45,46]. In the current study, personal contacts known to engage in Chemsex were invited to participate and to distribute study invitations across their social circles/networks (e.g., Chemsex-related WhatsApp or Telegram groups). Additionally, sexual health clinics, charities, and professional organisations dedicated to LGBTQ+ health were also invited to promote the study via their regular communication channels.

### 2.3. Data Collection and Storage

All data were handled according to UK General Data Protection Regulations. The data collection period continued between December 2023 and February 2024. Participant responses were collected anonymously using Microsoft Forms linked to a secure Microsoft SharePoint server administered by the University of Wolverhampton. No identifiable information was collected during the study. The final dataset and working files were password-protected and stored safely on institutional OneDrive cloud storage and personal encrypted drives.

### 2.4. Measures

Anxiety and depression were measured using the Hospital Anxiety and Depression Scale (HADS). The HADS questionnaire is one of the most widely employed validated tools for identifying potential cases of anxiety and depression, as well as for assessing symptom severity in various populations [47]. The HADS questionnaire is divided into two subscales: (1) the anxiety subscale (HADS-A) and (2) the depression subscale (HADS-D), each containing seven intermingled questions. Respondents provide answers on a four-point scale (0–3), resulting in a score range of 0 to 21. Scores of up to 7 points on either subscale are considered within the normal range. Scores of 8 and above on either subscale indicate probable cases of the respective states. Scores between 8 and 10 suggest the presence of the respective state, or ‘borderline abnormal’ state, while scores of 11 or higher indicate a clinically significant mood/anxiety disorder or ‘abnormal state’ [48].

To assess the frequency of engagement in Chemsex and the types of drugs/use behaviours, the participants were asked three questions: (1) ‘How often you participate in Chemsex?’; (2) ‘Which substances do you use during a Chemsex session?’; and (3) ‘Do you ever slam/inject?’. The selection of these questions was guided by previous studies [5,37,49]. Socio-demographic data was collected at the individual level (i.e., age, sexual orientation, and ethnicity). In addition, three questions at the socio-economic level regarding the highest attained level of education, employment status, and language were also included. The selection of those questions was partially based on the UK Office for National Statistics’ guidelines.

### 2.5. Data Analysis 

Owing to the small sample size, we were restricted to descriptive analysis only. To provide an overview of participation in Chemsex, responses to the question “How often do you participate in Chemsex?” were categorized as “weekly”, “bi-weekly”, “monthly”, and “less often”. Consequently, participants were categorized into groups including “non-cases”, “probable cases”, “borderline abnormal cases”, and “abnormal cases” based on the HADS cut-off criteria. The frequencies of each consumer drug were categorized under distinct headings, including “Chemsex-associated substances”, “additional substances”, “erectile dysfunction medications”, and “slamming”. Values for each consumer drug were tabulated across the four categories of Chemsex participation, and a chi-squared test of proportions was applied to data which met the minimum assumption for the validity of the test (i.e., a frequency of five or above in each cell).

## 3. Results

### 3.1. Socio-Demographic Profile 

We received a total of 147 responses to our survey. Six respondents (4.1%) did not complete the entire survey and were excluded. Consequently, 141 individuals (95.9%) were included in the report. 

The socio-demographic profile of the participants is presented in Table 1. The majority of participants identified as gay, resided in the London area, and were of white ethnicity. The age distribution showed a notable concentration within the 26–41 age bracket. A noteworthy proportion of participants had attained at least an undergraduate university degree, and most of the participants were actively employed. A considerable proportion reported English as their first language.

### 3.2. Participation in Chemsex

Among the 141 participants included in the analysis, 38 (27.0%) reported engaging in Chemsex on a weekly basis, while 34 (24.1%) engaged every other week, 33 (23.4%) participated monthly, and 36 (25.5%) participated less frequently than monthly.

Table 2 presents the use of specific consumer drugs during Chemsex. GHB/GBL emerged as one of the most frequent substances (77.3%), followed by synthetic cathinones (e.g., mephedrone) (64.5%) methamphetamine (47.5%), whereas consumption of ketamine and cocaine was less common (27.7% and 23.4%, respectively). In addition to Chemsex-associated substances, alkyl nitrates (poppers) were reported by 84 (59.6%) participants, while 36 (25.5%), 33 (23.4%), and 21 (14.9%) reported the use of ecstasy/MDMA, alcohol, and cannabis, respectively. Isolated instances of lysergic acid diethylamide (LSD)/psilocybin and amphetamine use were also noted.

A significant temporal association was detected between methamphetamine use and the frequency of participation in Chemsex according to the Chi-square test (*p* = 0.02). Specifically, among the 67 methamphetamine users, 28 individuals (41.8%) reported weekly consumption. Moreover, while intravenous drug use was less prevalent (17.0%), it was more frequently reported among individuals engaging in weekly Chemsex (54.2%). Conversely, it was least common among both monthly Chemsex users and those engaging less frequently, reported by only 3 individuals (12.5%) in both temporal categories. 

### 3.3. Prevalence of Anxiety and Depression 

As presented in Table 3, 70 individuals (49.9%) met the criteria for anxiety. Of those, 25 (35.7%) exhibited borderline abnormal scores, while 45 (64.3%) displayed abnormal scores, and a significant association was observed among participants identified as abnormal cases (*p* = 0.02). Specifically, the most severe outcomes were identified among individuals who engaged in Chemsex on a weekly and bi-weekly basis, indicating a potential dose-response relationship between more frequent Chemsex participation and increased severity of anxiety. The prevalence of probable depression was comparatively lower, with only 38 individuals (27.0%). Of those, 25 (54.4%) demonstrated borderline abnormal scores, while 13 (34.2%) exhibited abnormal scores. Notably, individuals engaging in weekly Chemsex scored the highest on the depressive scale. Significant associations were detected between Chemsex participation and the number of participants categorized as probable cases (*p* = 0.03).

## 4. Discussion

In our study, we noted a high frequency of casual Chemsex participation, where more than half of the participants engaged in Chemsex at least twice a month. Almost half of the participants met the HADS cut-off criteria for probable anxiety, with the most severe cases observed among those engaging in weekly Chemsex. While the limited data precluded us from establishing significance in associations between Chemsex frequency and borderline abnormal cases of anxiety, a significant relationship was established between more frequent Chemsex participation and higher severity of anxiety scores. The prevalence of depression was comparatively lower than anxiety, although significant associations were also observed between Chemsex participation and probable and abnormal cases of depression, suggesting a potential association between frequent Chemsex engagement and elevated depression scores. Our findings further complement the evident relationship between drug consumption patterns and an increased susceptibility to mental health risks amongst Chemsex users reported in the extant literature [34,35,36,37,50,51]. 

The observed phenomena may be partially attributed to the compounding influences of social isolation, stigma, and maladaptive coping strategies [32,52,53,54,55]. Chemsex users frequently fail to acknowledge the problematic nature of their substance use [53,55,56,57], with many in denial about their loss of control, especially with methamphetamine use, which increases their vulnerability [53,54]. Their behaviours often mirror those observed in other addictive disorders, including the development of physiological tolerance to the substances, impaired impulse control regarding consumption, and the prioritization of Chemsex over other important aspects of life [21,53,54,55,56,57]. Chemsex has been documented to often being practiced intermittently, with bouts of heavy usage interspersed with episodes of abstinence [21,58,59,60]. Increased engagement in Chemsex may precipitate social isolation, as individuals who prioritise those activities expose themselves to a constriction of social networks and detachment from non-Chemsex-using support systems [53,59,60]. The homophobia experienced by some participants in the extant literature and the difficulties in fully developing their sexuality may also contribute to the practice [53,59]. Such isolation, compounded by the internalised homophobia and societal stigma around Chemsex, may induce profound feelings of guilt and shame following a Chemsex episode, thereby perpetuating or exacerbating preexisting mental health vulnerabilities [53,54,55]. The literature often describes participants’ experiences of physical fatigue, emotional instability, mood swings, apathy, and feelings of loneliness, which further compounds the issue [53,54,55]. The fear of being judged, particularly in healthcare settings, may prevent individuals from seeking help, causing them to hide their drug use. As a consequence, Chemsex may serve as a maladaptive coping mechanism, wherein it provides a temporary relief from underlying emotional distress and a sense of safety and acceptance [54,55,57]. For some, it has been described as a space where they do not have to explain their HIV status and can avoid feelings of shame and guilt [53].

With regards to Chemsex-associated substance use in the present study, GHB/GBL, synthetic cathinones (markedly mephedrone), and methamphetamine were amongst the most frequently reported substances consumed. Furthermore, methamphetamine use showed a significant association with the frequency of participation in Chemsex, particularly among those who reported weekly usage. According to the literature, individuals with methamphetamine use disorder have significantly higher odds of depression compared to those to those who do not use this substance [50,51]. A 2021 Australian study involving a community sample of 725 individuals using methamphetamine showed that more than half of the participants were classified as experiencing moderate-to-severe anxiety and/or depression (13.7% anxiety and 37.7% depression) [50]. Furthermore, a recent meta-analysis of data from 14 studies found significant associations between methamphetamine exposure and increased odds of depression in both cross-sectional studies (OR =  1.66, 95% CI = 1.34 to 2.05) and longitudinal studies (OR =  1.18, 95% CI = 1.08 to 1.28) [51]. The use of intravenous drugs during Chemsex in the present study was also common, whereby weekly participants engaged in this form of drug administration more frequently. Indeed, previous studies have recognized Slamsex as a significant risk for worse mental health outcomes [40,41,42], which highlights the importance of considering not only the types of drugs used, but also the specific behaviours and practices associated with Chemsex in understanding its potential impact on individual health and well-being. 

Considering the nature of the substances used during Chemsex, their direct physical effects cannot be ignored. It is noteworthy that Chemsex sessions often involve prolonged periods of wakefulness and disrupted sleep patterns [1,2], and sleep deprivation is a well-established risk factor for mood disorders, including depression and anxiety [61]. Most Chemsex drugs, including methamphetamine, GHB/GBL, and mephedrone, can exert neurotransmitter system dysregulation via direct effects on those systems, particularly the dopamine, serotonin, and norepinephrine systems [62,63,64]. These neurotransmitter systems play fundamental roles in reward processing and mood regulation, as well as the regulation of stress [65]. Frequent use of Chemsex substances can lead to depletion and dysregulation of these systems, contributing to mood instability, anxiety, and depressive symptoms [62,63,64,65]. Simultaneously, some substances, particularly methamphetamine, have known neurotoxic effects, potentially causing long-term damage to brain regions involved in emotional processing [62]. Furthermore, chronic exposure to methamphetamine can alter the brain circuitry involved in reward, motivation, and emotional regulation [66]. 

Considering these findings, targeted interventions aimed at reducing the frequency of Chemsex or mitigating its negative impact on mental health would be reasonable. These could include harm reduction strategies such as providing access to mental health services, substance use treatment programs, and peer support groups tailored to the unique needs of individuals. Digital platforms show promising opportunities for harm reduction, with studies indicating improvements in self-efficacy, the avoidance of risky sexual behaviours, and an increased uptake of HIV testing among MSM [67,68]. Practical harm reduction strategies include the provision of harm-reduction kits through tele-health consultations or mobile platforms. These kits, delivered discretely, aim to minimise the adverse effects of chemsex and promote safer sexual practices to ensure that individuals feel safe and less exposed to legal or social repercussions [69,70]. Integrating harm reduction approaches into existing health services, such as psychotherapy, sexology, and addiction counselling, is also imperative [69,71]. In particular, approaches involving motivational interviewing and therapeutic education, delivered through multidisciplinary teams, can serve as effective starting points [69,71]. Furthermore, mental health clinic engagement has been linked to increased intention to reduce chemsex behaviors [69,72]. Finally, targeted training in LGBT venues to address drug overdoses has demonstrated a positive impact on reducing drug use and sexual risk behaviors among Chemsex users [69,73].

Despite providing novel insights, this study has several important limitations that should be noted. First, the small sample size may limit the generalizability of our findings, as it may not accurately represent the broader population, which is further complicated that our cohort included individuals residing predominantly in the London area. To mitigate the potential recruitment challenges, we chose to employ a non-probability sampling, to which many limitations can be attributed. In our case, self-selection bias could potentially influence the study demographic, as those self-selected may not represent the wider population. Additionally, the cross-sectional design of this study precludes us from establishing causality or exploring potential underlying mechanisms between Chemsex and mental health. While this study did not seek to establish causality, future longitudinal research incorporating comprehensive assessments of both the patterns of substance use and mental health status to elucidate the temporal dynamics and underlying pathways linking Chemsex to anxiety and depression is warranted. Additionally, research employing qualitative methodologies is recommended to gain nuanced understanding of these contributing factors.

## 5. Conclusions

Our investigation into the psychological well-being of Chemsex users indicated a high prevalence of anxiety and depression, supporting existing literature that highlights mental health challenges experienced by Chemsex users. The present findings indicate that substance use, particularly when engaged in frequently or compulsively, may serve as a maladaptive coping mechanism for underlying emotional issues, contributing to worsened mental health outcomes. These results also underline the importance of considering the frequency of Chemsex engagement. The observed variations in anxiety and depression scores across different frequencies of Chemsex engagement highlight the possible interplay between substance use patterns and mental health outcomes. Individuals participating in Chemsex on a weekly basis exhibited significantly higher levels of anxiety and depression compared to those engaging less frequently. This may suggest a potential dose-response relationship, wherein increased exposure to substances used during Chemsex may exacerbate psychological distress. However, this may also indicate that individuals with who experience higher anxiety/depression levels are more likely to engage in Chemsex more frequently. Therefore, it is important to acknowledge the complexity of these associations. Factors such as social support, stigma, access to healthcare, and individual resilience may modulate the impact of Chemsex on mental health. Future research should further explore these aspects.

## Figures and Tables

**Table 1:** Key characteristics of the study participants (N = 141).

	N	%
Residence		
London	116	83.2
Other	25	16.8
Age group		
18–21	1	0.7
22–25	8	5.7
26–29	26	18.4
30–33	32	22.7
34–37	35	24.8
38–41	19	13.5
42–45	8	5.7
46–49	4	2.8
50+	8	5.7
Sexual orientation		
Gay	131	92.9
Bisexual	10	7.1
Ethnicity		
White	108	76.6
Mixed	10	7.1
Black	7	5.0
South/Southeast Asian	4	2.8
Arab/Middle Eastern	3	2.1
East Asian	1	0.7
Other/Not specified	8	5.7
Highest level of education		
Primary school	2	1.4
Secondary school	10	7.1
Sixth form/college diploma	25	17.7
At least undergraduate university degree, of which:	103	73.1
Undergraduate university degree only	50	35.5
Postgraduate diploma/master’s degree	39	27.7
Higher research or practice degree (e.g., PhD, MD)	14	9.9
No formal qualification	1	0.7
Working status		
Any employment, of which:	130	92.2
Full-time	87	66.9
Self-employed	29	22.3
Part-time	14	10.8
Unemployed	9	6.4
Student	2	1.4
First language		
English	96	58.1
Other/Not otherwise specified	45	31.9

**Table 2:** Substances used in a Chemsex setting. Gamma hydroxybutyrate/gamma-butyrolactone (GHB/GBL), synthetic cathinones, and methamphetamine were the most common substances used in this study sample. Methamphetamine and slamming were most common amongst weekly users.

	Overall (N = 141)	Weekly (n = 38)	Bi-Weekly (n = 34)	Monthly (n = 33)	Less Often (n = 36)	Chi-Square Test
	% (N)	% (n)	% (n)	% (n)	% (n)	*p*-Value
Chemsex-associated drugs						
GHB/GBL	77.3 (109)	31.2 (34)	28.4 (31)	23.9 (26)	16.5 (18)	0.15
Synthetic cathinones (mephedrone)	64.5 (91)	26.4 (24)	28.6 (26)	26.4 (24)	18.7 (17)	0.56
Methamphetamine	47.5 (67)	41.8 (28)	16.4 (11)	20.9 (14)	20.9 (14)	0.02
Ketamine *	27.7 (39)	33.3 (13)	10.3 (4)	23.1 (9)	33.3 (13)	n/a
Cocaine *^#^	23.4 (33)	12.1 (4)	18.2 (6)	39.4 (13)	30.3 (10)	n/a
Additional substances						
Alkyl nitrates (poppers)	59.6 (84)	22.6 (19)	21.4 (18)	22.6 (19)	33.3 (28)	0.37
Ecstasy/MDMA	25.5 (36)	25.0 (9)	8.3 (3)	30.6 (11)	36.1 (13)	n/a
Alcohol	23.4 (33)	12.1 (4)	21.2 (7)	24.2 (8)	42.4 (14)	n/a
Cannabis	14.9 (21)	23.8 (5)	23.8 (5)	9.5 (2)	42.9 (9)	n/a
LSD/psilocybin	0.0 (1)	100 (1)	n/a	n/a	n/a	n/a
Amphetamines	0.0 (1)	n/a	100 (1)	n/a	n/a	n/a
Erectile dysfunction medications (e.g., Viagra)	70.2 (99)	28.3 (28)	27.3 (27)	26.3 (26)	18.2 (18)	0.47
Slamming	17.0 (24)	54.2 (13)	20.8 (5)	12.5 (3)	12.5 (3)	n/a

Annotations: *, recently classified as Chemsex substances in the UK; ^#^, includes crack cocaine. Abbreviations: LSD, Lysergic acid diethylamide.

**Table 3:** Classification of anxiety and depression amongst study participants.

	Overall (N = 141)	Weekly (n = 38)	Bi-Weekly (n = 34)	Monthly (n = 33)	Less Often (n = 36)	Chi-Square Test
	% (n)	% (n)	% (n)	% (n)	% (n)	*p*-Value
Anxiety						
Non-cases (HADS-A ≤ 7)	51.1 (71)	16.9 (12)	26.8 (19)	19.7 (14)	36.6 (26)	
Any cases (HADS-A ≥ 8)	49.9 (70)	37.1 (26)	21.4 (15)	27.1 (19)	14.3 (10)	0.05
Borderline abnormal (HADS-A 8–10) *	35.7 (25)	24.0 (6)	28.0 (7)	36.0 (9)	12.0 (3)	n/a
Abnormal (HADS-A 11–21) *	64.3 (45)	44.4 (20)	17.8 (8)	22.2 (10)	15.6 (7)	0.02
Depression						
Non-cases (HADS-D ≤ 7)	73.0 (103)	20.4 (21)	26.2 (27)	23.3 (24)	30.1 (31)	
Any cases (HADS-D ≥ 8)	27.0 (38)	44.7 (17)	18.4 (7)	23.7 (9)	13.2 (5)	0.03
Borderline abnormal (HADS-D 8–10) *	65.8 (25)	36.0 (9)	24.0 (6)	28.0 (7)	12.0 (3)	n/a
Abnormal (HADS-D 11–21) *	34.2 (13)	61.5 (8)	0.7 (1)	15.4 (2)	15.4 (2)	n/a

* % of probable cases.

## Data Availability

Data used in this research can be made available upon request from the corresponding author.
